# At Last

**Published:** 1880-06

**Authors:** 


*At Last.*—The papers of June 10th give an account of a "raid" made on a number of so-called medical colleges in Philadelphia which for years past have scattered "bogus" medical diplomas from that city over this and foreign lands. We believe that the fraudulent dental diplomas issued in the same city are from this firm; if this is true, this raid will prove as great a blessing to the dental as to the medical profession. According to the reports they have been selling diplomas "for a consideration" to every part of the world, but especially to Germany. Dr. John

Buchanan, the alleged chief offender, has been committed by the United States commissioner in default of $10,000 bail on a charge of using the mails in connection with the business. The final exposure and arrest were due to the enterprise and public spirit of Mr. John Morris, city editor of the Philadelphia *Record*, who accomplished what the legal authorities have repeatedly failed to achieve. Recently the matter has been brought conspicuously before the public by some correspondence of President Andrew D. White, of Cornell University, at present United States minister to Germany. Mr. White resented some allusions in a popular farce to quacks created by American ready-made diplomas, but at the same time admitted and deprecated this traffic in spurious degrees. The matter was, however, exposed and ventilated several years ago by the New York *Nation*. On that occasion one of the persons accused in the present case proved that he represented a regularly chartered college, with authority to grant diplomas, and threatened a suit for libel. In the course of the controversy which ensued it came out that Germany's skirts were not clean in regard to these same practices about which its people bring such loud accusations against this country. It was proved that the great University of Leipsic had been in the habit of vending its diplomas to liberal bidders, and three cases were cited of such diplomas granted within the year. The practice, however, has been entirely broken up in Germany, and it is to be hoped that the present exposures and prosecutions will end in breaking it up here also. H.

